# Metabolic Characterization of Antifolate Responsiveness and Non-responsiveness in Malignant Pleural Mesothelioma Cells

**DOI:** 10.3389/fphar.2018.01129

**Published:** 2018-10-12

**Authors:** Yuzo Sato, Shiori Matsuda, Ami Maruyama, Joji Nakayama, Tomoyuki Miyashita, Hibiki Udagawa, Shigeki Umemura, Kazuyoshi Yanagihara, Atsushi Ochiai, Masaru Tomita, Tomoyoshi Soga, Katsuya Tsuchihara, Hideki Makinoshima

**Affiliations:** ^1^Tsuruoka Metabolomics Laboratory, National Cancer Center, Tsuruoka, Japan; ^2^Shonai Regional Industry Promotion Center, Tsuruoka, Japan; ^3^Systems Biology Program, Graduate School of Media and Governance, Keio University, Fujisawa, Japan; ^4^Institute for Advanced Biosciences, Keio University, Tsuruoka, Japan; ^5^Division of Translational Research, Exploratory Oncology Research and Clinical Trial Center, National Cancer Center, Kashiwa, Japan; ^6^Department of Thoracic Oncology, National Cancer Center Hospital East, Kashiwa, Japan; ^7^Division of Biomarker Discovery, Exploratory Oncology Research and Clinical Trial Center, National Cancer Center, Kashiwa, Japan; ^8^Exploratory Oncology Research and Clinical Trial Center, National Cancer Center, Kashiwa, Japan

**Keywords:** tumor metabolism, mesothelioma, antifolate therapy, purine, pyrimidine

## Abstract

Antifolates are a class of drugs effective for treating malignant pleural mesothelioma (MPM). The majority of antifolates inhibit enzymes involved in purine and pyrimidine synthesis such as dihydrofolate reductase (DHFR), thymidylate synthase (TYMS), and glycinamide ribonucleotide formyltransferase (GART). In order to select the most suitable patients for effective therapy with drugs targeting specific metabolic pathways, there is a need for better predictive metabolic biomarkers. Antifolates can alter global metabolic pathways in MPM cells, yet the metabolic profile of treated cells has not yet been clearly elucidated. Here we found that MPM cell lines could be categorized into two groups according to their sensitivity or resistance to pemetrexed treatment. We show that pemetrexed susceptibility could be reversed and DNA synthesis rescued in drug-treated cells by the exogenous addition of the nucleotide precursors hypoxanthine and thymidine (HT). We observed that the expression of pemetrexed-targeted enzymes in resistant MPM cells was quantitatively lower than that seen in pemetrexed-sensitive cells. Metabolomic analysis revealed that glycine and choline, which are involved in one-carbon metabolism, were altered after drug treatment in pemetrexed-sensitive but not resistant MPM cells. The addition of HT upregulated the concentration of inosine monophosphate (IMP) in pemetrexed-sensitive MPM cells, indicating that the nucleic acid biosynthesis pathway is important for predicting the efficacy of pemetrexed in MPM cells. Our data provide evidence that may link therapeutic response to the regulation of metabolism, and points to potential biomarkers for informing clinical decisions regarding the most effective therapies for patients with MPM.

## Introduction

Malignant pleural mesothelioma is a locally invasive and rapidly fatal malignancy linked to asbestos exposure (Liu et al., 2017; Yap et al., 2017). MPM develops in the pleural cavity and is highly resistant to a number of therapeutics, with prognosis of patients remaining poor (Creaney and Robinson, 2017; Liu et al., 2017; Scherpereel et al., 2018). A combination of pemetrexed (PMX, also called Alimta or LY231514) and cisplatin has been the first line chemotherapy regimen for more than a decade (Vogelzang et al., 2003; Scagliotti et al., 2008; Liu et al., 2017). Pemetrexed is an antifolate that is able to simultaneously inhibit the synthesis of purines and pyrimidines (Shih et al., 1997). In clinical use, treatment with pemetrexed plus cisplatin and vitamin supplements resulted in superior survival, time to progression, and response rates compared to treatment with cisplatin alone in patients with MPM(Vogelzang et al., 2003; Scagliotti et al., 2008). Pemetrexed and its polyglutamated derivatives inhibit thymidylate synthase (*TYMS*), dihydrofolate reductase (*DHFR*), and glycinamide ribonucleotide transformylase (*GART*), all of which are involved in the *de novo* biosynthesis of thymidine and purine nucleotides (Shih et al., 1997;Yap et al., 2017). Antimetabolite agents, including pemetrexed, induce an imbalance in the cellular nucleotide pool and inhibit nucleic acid biosynthesis that results in arresting the proliferation of tumor cells and inducing cell death(Zhao and Goldman, 2003; Yap et al., 2017).

The discovery of oncogenic driver mutations has allowed the identification of druggable targets and development of new therapies using small molecule tyrosine kinase inhibitors (TKI) aimed at the relevant patient populations (Irmer et al., 2007; Levitzki, 2013; Hylebos et al., 2016). Comprehensive genomic analysis of MPM identified recurrent mutations, gene fusion and splicing alterations (Bueno et al., 2016). Through integrated analyses, alterations were identified in Hippo, mTOR, histone methylation RNA helicase and TP53 signaling pathways in MPM (Bueno et al., 2016). Other studies demonstrated that the most frequent genetic variations clustered into two main pathways (Hylebos et al., 2016). The first altered pathway was the TP53/DNA repair pathway with genetic variations in *TP53, BAP1* and *CDKN2A* genes, and the second pathway was the PI3K/AKT pathway, with genetic variations in *KIT, KDR, PIK3CA* and *NF2* genes, respectively (De Rienzo et al., 2016; Hylebos et al., 2016). However, there has been a paucity of new actionable mutations in MPM as drug targets.

Accumulating evidence shows that genetic mutations in cancer-driver genes, tumor suppressors, and amplified oncogenes are linked to specific alterations in metabolic pathways in cancer cells, involving proteins such as isocitrate dehydrogenase (IDH), fumarate hydratase (FH), MYC, K-RAS and BRAF (Levine and Puzio-Kuter, 2010; Cairns et al., 2011; Cheong et al., 2012; Dejure and Eilers, 2017; Palm and Thompson, 2017). The Warburg effect, the phenomenon in which cancer cells exhibit intense glucose consumption with production of lactate despite abundant oxygen availability, has been recognized since the 1930s (Vander Heiden et al., 2009; Lunt and Vander Heiden, 2011; Soga, 2013). Genetic mutations in tumor cells might cause several unique metabolic phenotypes that are critical for cancer cell proliferation in MPM. The frequent loss of CDKN2A (at 9p21) in MPM typically includes the homozygous co-deletion of MTAP (Illei et al., 2003). Specifically, MTAP catalyzes the reversible phosphorylation of MTA to the purine adenine and 5-methylthioribose-1-phosphate and PRMT5 inhibition induced metabolic vulnerability (Kryukov et al., 2016; Mavrakis et al., 2016; Yap et al., 2017). The MTAP protein plays a crucial role in polyamine metabolism involving salvage of adenosine and methionine from the substrate MTA (Bertino et al., 2011; Makinoshima et al., 2018).

One-carbon metabolism involving the folate and methionine cycle integrates carbon units from amino acids and generates diverse outputs, such as the biosynthesis of nucleotides, lipids and proteins in cancer cells (Yang and Vousden, 2016; Ducker and Rabinowitz, 2017; Newman and Maddocks, 2017). Glycine can be utilized for *de novo* purine biosynthesis by two mechanisms: direct incorporation into the purine backbone or further oxidation by the glycine cleavage system (GCS) to yield one-carbon units for nucleotide synthesis and cellular methylation reactions (Amelio et al., 2014; Newman and Maddocks, 2017). The GCS has also been implicated in cell transformation and tumorigenesis (Zhang et al., 2012). Given the high proliferation rate of cancer cells and the requirement of nucleotides for proliferation, cancer cells have a large demand for one-carbon units for nucleotide synthesis (Yang and Vousden, 2016; Ducker and Rabinowitz, 2017; Newman and Maddocks, 2017). To this day, chemical variants of these initial folate antagonists such as methotrexate and pemetrexed constitute a major class of cancer chemotherapy agents and are used as frontline chemotherapy for diverse cancers (Zhao and Goldman, 2003).

In this paper, we characterized the metabolic features of mesothelioma using a non-targeted metabolic profiling strategy based on capillary electrophoresis-mass spectrometry (CE/MS). MPM cell lines were categorized into two groups according to their susceptibility to pemetrexed treatment. Using end product rescue, we showed that treatment with pemetrexed mainly targets pyrimidine biosynthesis rather than purine biosynthesis in MPM cells. We also demonstrated a metabolic response including one-carbon cycle against pemetrexed treatment in sensitive or resistant MPM cells. Our results link the antifolate therapeutic response to the regulation of metabolism and imply that the levels of glycine and IMP are potential biomarkers that may inform the clinical utility of specific targeted therapies to treat patients with MPM.

## Experimental Procedures

### Materials

MPM an aggressive malignancy affecting pleural surfaces, is divided into three main histological subtypes. The epithelioid and sarcomatoid subtypes are characterized by cuboid and fibroblastoid cells, respectively. The biphasic subtype contains a mixture of both. Commercially available cell lines were purchased from the American Type Culture Collection (ATCC), which included MSTO-211H (biphasic), NCI-H28 (epithelial), NCI-H226 (epithelial), NCI-H2052 (epithelial) and NCI-H2452 (epithelial). 3 MPM cell lines (TCC-MESO1 (epithelial), TCC-MESO2 (epithelial), and TCC-MESO3 (biphasic)) were established from Japanese patients with MPM and some of the biological characteristics were analyzed in a previous report (Yanagihara et al., 2010). All cell lines were cultured in RPMI-1640 supplemented with 10% FBS. RPMI-1640 (R8758), glucose minus RPMI-1640 (R1383), phosphate buffered saline (PBS), hypoxanthine, thymidine, 2-deoxy-D-glucose (2DG) and MTA were purchased from Sigma–Aldrich (St. Louis, MO, United States). Fetal bovine serum (FBS) was purchased from Biowest (Nuaille, France). Dimethyl sulfoxide (DMSO) and glucose were purchased from Wako Pure Chemicals Industries (Osaka, Japan). Pemetrexed was purchased from Selleck (Houston, TX, United States). Cell Counting Kit-8 was purchased from Dojindo Laboratories (Kumamoto, Japan). FluxPak XF24 assay pack and XF glycolysis stress test kit was purchased from Seahorse Bioscience (North Billerica, MA, United States). Propidium iodide (PI) was purchased from Thermo Fisher Scientific (Waltham, MA, United States). Mini-PROTEAN TGX Precast Gel, Trans-Blot Turbo Transfer System and Trans-Blot Turbo Transfer Pack were purchased from Bio-Rad Laboratories, Inc. (Hercules, CA, United States). Primary antibodies specific for DHFR, GART and TYMS were purchased from Abcam (Cambridge, United Kingdom) and primary antibody to phospho-RB1 (pRB1), RB1, p21 and GAPDH were purchased from Cell Signaling Technologies (Danvers, MA, United States). The peroxidase-linked secondary antibodies for WB, HRP-linked sheep anti-mouse IgG and donkey anti-rabbit IgG, were purchased from GE Healthcare Biosciences (Pittsburgh, PA, United States). Oligomycin was purchased from Merck Millipore (Darmstadt, Germany). SYBR Premix Ex Taq and primers were purchased from TaKaRa Bio (Shiga, Japan). Ribonuclease A (RNase A) was purchased from Roche Applied Science (Penzberg, Germany) and contaminating DNase was inactivated at 80°C for 30 min.

### Cell Survival Assay and Proliferation Assay

MPM cells were seeded in RPMI-1640 containing various concentrations of pemetrexed in 96-well cell culture plates. After 72 h of incubation, cell viability was analyzed using a WST-8 assay using the Cell Counting Kit-8. The pemetrexed concentration against the percent of cell survival was plotted using all MPM cell lines. The IC_50_ values were calculated using Graph Pad Prism 7 software (GraphPad Software, Inc., La Jolla, CA, United States). For rescue experiments, MPM cells were cultured in complete medium supplemented with 100 μM hypoxanthine alone, 16 μM thymidine alone or mixed hypoxanthine plus thymidine in the presence of pemetrexed.

### Cell Cycle Analysis

Cell cycle distribution was analyzed by flow cytometry. PBS, pemetrexed or pemetrexed + HT treated cells were harvested, washed twice with PBS, and fixed in 70% ethanol overnight at -20°C. Fixed cells were harvested and washed twice with PBS, incubated with 1 ml of PBS containing 40 μg/ml propidium iodide and 100 μg/ml RNase A for 30 min at 37°C. Stained cells were analyzed using a Becton Dickinson FACSMelody instrument (Franklin Lakes, NJ, United States). FlowJo software (FlowJo, LLC, Ashland, OR, United States) was used for data analysis and generation of graphs. Single cell populations were gated and the percentage of cells in the various stages of the cell cycle (G0/G1, S, and G2/M phases) was determined by the Watson Model.

### Measurement of ECAR and OCR

ECAR and OCR were measured with a XF glycolysis stress test kit according to the manufacturer’s instructions (Seahorse Bioscience, Agilent). In brief, 4.5 × 10^4^ cells were plated onto XF24 plates in RPMI-1640 (10% FBS, 2 mM glutamine) and incubated at 37°C, 5% CO_2_ overnight. Cells were washed with assay medium (minus glucose and unbuffered RPMI-1640 (SIGMA R1383)), replaced with assay medium, and then placed at 37°C in a CO_2_-free incubator for 30 min. ECAR and OCR were monitored using a Seahorse Bioscience XF24 Extracellular Flux Analyzer over time and each cycle consisted of 3 min mixing, 3 min waiting, and 3 min measuring. Glucose, oligomycin and 2DG were diluted into XF24 media and loaded into the accompanying cartridge to achieve final concentrations of 10 mM, 5 μM, and 100 mM respectively. Injections of the drugs into the medium occurred at the time points specified.

### Western Blotting

Cells were lysed in CST lysis buffer on ice for 2 min and centrifuged at 15,000 × *g* for 10 min. The protein content of supernatants was measured by BCA assay (Pierce). Equivalent amounts of protein samples were separated via 4–20% SDS/PAGE, transferred to PVDF membranes, and incubated overnight with primary antibodies (1:1000 dilution). The primary antibodies used in this study are listed in the materials. ECL anti-rabbit IgG HRP-linked whole antibody (1:10,000; GE Healthcare) was used as secondary antibody. Signals were detected using ECL Western Blotting Detection reagent (GE Healthcare) and FUSION FX (VILBER, France). The intensity of bands was quantified using Fusion Capt Advance FX7 software.

### Quantitative RT-PCR

Cells were washed with PBS and total RNA from the MPM cell lines was isolated with TRIzol Reagent (Invitrogen). Complementary DNA (cDNA) was synthesized using the SuperScript VILO cDNA synthesis kit (Invitrogen). Primers for metabolic genes were purchased from TaKaRa Bio (Japan). Real-time RT–PCR was carried out with specific primers and a QuantStudio 3 Thermo Fisher Scientific (Waltham, MA, United States). GAPDH was used for normalization as control and the relative quantitation value compared to the calibrator for that target is expressed as 2^-(Ct-Cc)^.

### Metabolite Measurements

Metabolic extracts were prepared from 1–5 × 10^6^ MPM cells with methanol containing internal standard solution (Human Metabolome Technologies; HMT, Inc., Tsuruoka, Japan) and analyzed using a capillary electrophoresis (CE)-connected electrospray ionization/time-of-flight mass spectrometry (ESI-TOFMS) and capillary electrophoresis tandem mass spectroscopy (CE-MS/MS) system (CARCINOSCOPE, Human Metabolic Technologies, Tsuruoka, Japan). Procedures for metabolite measurements were as previously described (Makinoshima et al., 2014, 2018). In brief, cells were washed twice in 5% mannitol solution and then treated with 800 μL of methanol and 550 μL of Milli-Q water containing internal standards (H3304-1002, HMT, Inc., Tsuruoka, Japan). The metabolite extract was transferred into a microfuge tube and centrifuged at 2,300 × *g* and 4°C for 5 min. Next, the upper aqueous layer was centrifugally filtered through a Millipore 5-kDa cutoff filter to remove proteins. The filtrate was centrifugally concentrated and resuspended in 50 μL of Milli-Q water for CE-MS analysis. Cationic compounds were analyzed in the positive mode of CE-TOFMS and anionic compounds were analyzed in the positive and negative modes of CE-MS/MS according to the methods developed by Soga and Heiger (2000) and Soga et al. (2002, 2003). To obtain peak information including *m/z*, migration time (MT), and peak area, detected peaks by CE-TOFMS and CE-MS/MS were extracted using automatic integration software (MasterHands, Keio University, Tsuruoka, Japan and MassHunter Quantitative Analysis B.04.00, Agilent Technologies, Santa Clara, CA, United States, respectively). Concentrations of metabolites were calculated by normalizing the peak area of each metabolite with respect to the area of the internal standard and by using standard curves obtained by three-point calibrations.

### Statistical Analyses

Unless otherwise indicated, results were reported as the mean ± SD. Statistical analyses were done by two-tailed Student’s *t*-test and *p*-values were indicated as ^∗^ < 0.05, ^∗∗^ < 0.01 and ^∗∗∗^ < 0.005. For metabolomic data analysis we used Welch *t*-test and *p*-values were indicated as ^∗^ < 0.05, ^∗∗^ < 0.01, and ^∗∗∗^ < 0.001.

## Results

### IC_50_ Measurements in 8 Mesothelioma Cell Lines Treated With Pemetrexed

**FIGURE 1:**
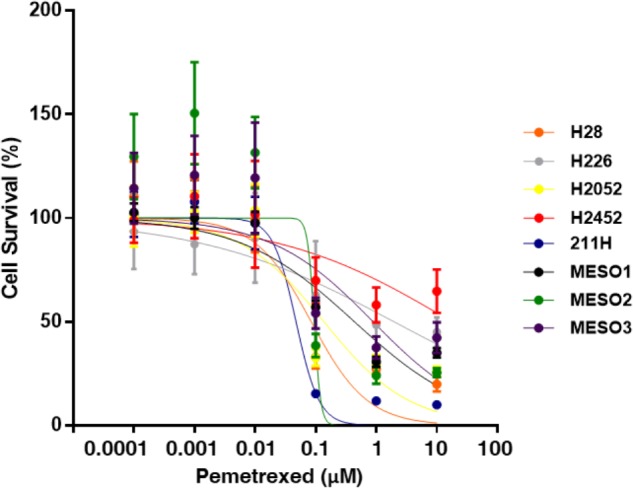
Pemetrexed treatment represses cell growth of MPM cells. WST-8 assay with pemetrexed. Cells were treated with the indicated concentration of pemetrexed for 72 h, and their viability was assessed by the WST-8 assay. The *%* viability data are shown as the mean ± standard deviation (SD) (*n* = 6). Error bars indicates the range of SD. Blue line: MSTO-211H, Orange line: NCI-H28, Gray line: NCI-H226, Yellow line: NCI-H2052, Red line: NCI-H2452, Black line: TCC-MESO1, Green line: TCC-MESO2, and Purple line: TCC-MESO3. The *in vitro* half maximal inhibitory concentration (IC_50_) of pemetrexed for the growth of MPM cell lines was calculated as the following values for each cell line: NCI-H28 = 84.1 nM, NCI-H226 = 1950 nM, NCI-H2052 = 135 nM, NCI-H2452 = >10,000 nM, MSTO-211H = 47.4 nM, TCC-MESO1 = 435 nM, TCC-MESO2 = 94.3 nM, and TCC-MESO3 = 883 nM.

We analyzed the *in vitro* sensitivity of MPM cell lines to the antifolate inhibitor pemetrexed through proliferation assays. MPM cell growth inhibition dose-response curves of pemetrexed are shown in **Figure 1**. The cytotoxic effect of pemetrexed on human MPM cells was analyzed using a WST-8 cell counting kit after 72 h of exposure. The IC_50_ value was defined as the dose of pemetrexed required to produce a 50% reduction in the viability of MPM cells. IC_50_ values were measured in 8 MPM cell lines after 72 h treatment with pemetrexed and found to range from 47.4 nM to ≥10,000 nM (**Figure 1**) as follows: MSTO-211H (47.4 nM), NCI-H28 (84.1 nM), NCI-H226 (1950 nM), NCI-H2052 (135 nM), NCI-H2452 (>10,000 nM), TCC-MESO1 (435 nM), TCC-MESO2 (94.3 nM), TCC-MESO3 (883 nM). We found that MPM cell lines could be categorized into two groups according to their susceptibility to pemetrexed treatment. In agreement with previous findings (Satoh et al., 2017), the NCI-H2452 cell line was resistant to pemetrexed treatment as compared to other MPM cell lines. MSTO-211H was highly sensitive to pemetrexed in the nanomolar range as compared to the pemetrexed-resistant cell line NCI-H2452 (**Figure 1**). For the rest of this paper, we utilized the MSTO-211H and TCC-MESO2 cell lines as pemetrexed-sensitive cells and the NCI-H2452 cell line as pemetrexed-resistant cells.

### End Product Reversal Studies of Pemetrexed in MPM Cells

**FIGURE 2:**
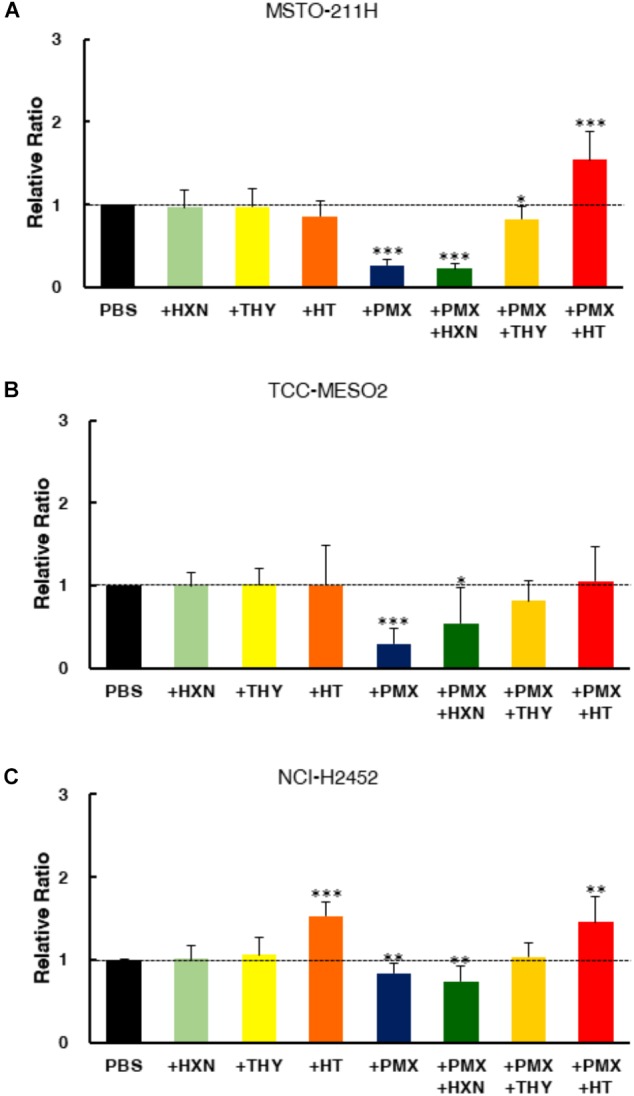
End product reversal analysis of pemetrexed in MPM cells. Cell proliferation was measured with the WST-8 assay in the presence of either nucleotide precursor alone (thymidine: THY, hypoxanthine: HXN), pemetrexed (PMX) alone, or PMX + nucleotide precursor. Two pemetrexed-sensitive cell lines, MSTO-211H **(A)** and TCC-MESO2 **(B)**, and a pemetrexed-resistant cell line NCI-H2452 **(C)** were used- Black: PBS, Light Green: 100 μM hypoxanthine (HXN), Light Yellow: 16 μM thymidine (THY), Orange: 100 μM hypoxanthine +16 μM thymidine (HT), Blue: Pemetrexed (PMX) 1 μM, Green: PMX 1 μM + 100 μM HXN, Yellow: PMX 1 μM + 16 μM THY, Red: PMX 1 μM + HT. Dotted line denotes the level of proliferation by PBS control set at 1.0. Error bars indicate *s.d.* (*n* = 6). ^∗^*P* < 0.05, ^∗∗^*P* < 0.01, ^∗∗∗^*P* < 0.005 vs. control by two-tailed Student’s *t*-test.

To confirm the target specificity of pemetrexed, we tested the ability of exogenous nucleotide precursors (hypoxanthine and thymidine) to rescue the antiproliferative effects of pemetrexed on either sensitive or resistant MPM cells (**Figure 2**). The cytotoxic effect of pemetrexed on human MPM cells was analyzed using the WST-8 cell counting kit after 72 h of exposure. The addition of either hypoxanthine (HXN) or thymidine (THY) did not change the cell proliferation rate as compared to PBS control (**Figures 2A–C**). Treatment with PMX significantly reduced cell growth in both sensitive and resistant MPM cells (**Figures 2A–C**). In agreement with previous findings (Shih et al., 1997), the addition of HXN partially rescued PMX cytotoxicity in TCC-MESO2 but had no effect for MSTO-211H and NCI-H2452 cell lines (**Figure 2**). Next, we found that the addition of THY nullified PMX cytotoxicity in TCC-MESO2 (**Figure 2B**) and NCI-H2452 (**Figure 2C**) cells, and partially in MSTO-211H cells (**Figure 2A**). The combination of hypoxanthine plus thymidine (+HT) completely rescued the growth repression induced by pemetrexed and the cell numbers here were significantly higher than those seen with PBS control in MSTO-211H and NCI-H2452 cells. These results demonstrate the target specificity of pemetrexed in MPM cells.

### Treatment of MPM Cells With Pemetrexed Alters Cell Cycle Populations

**FIGURE 3:**
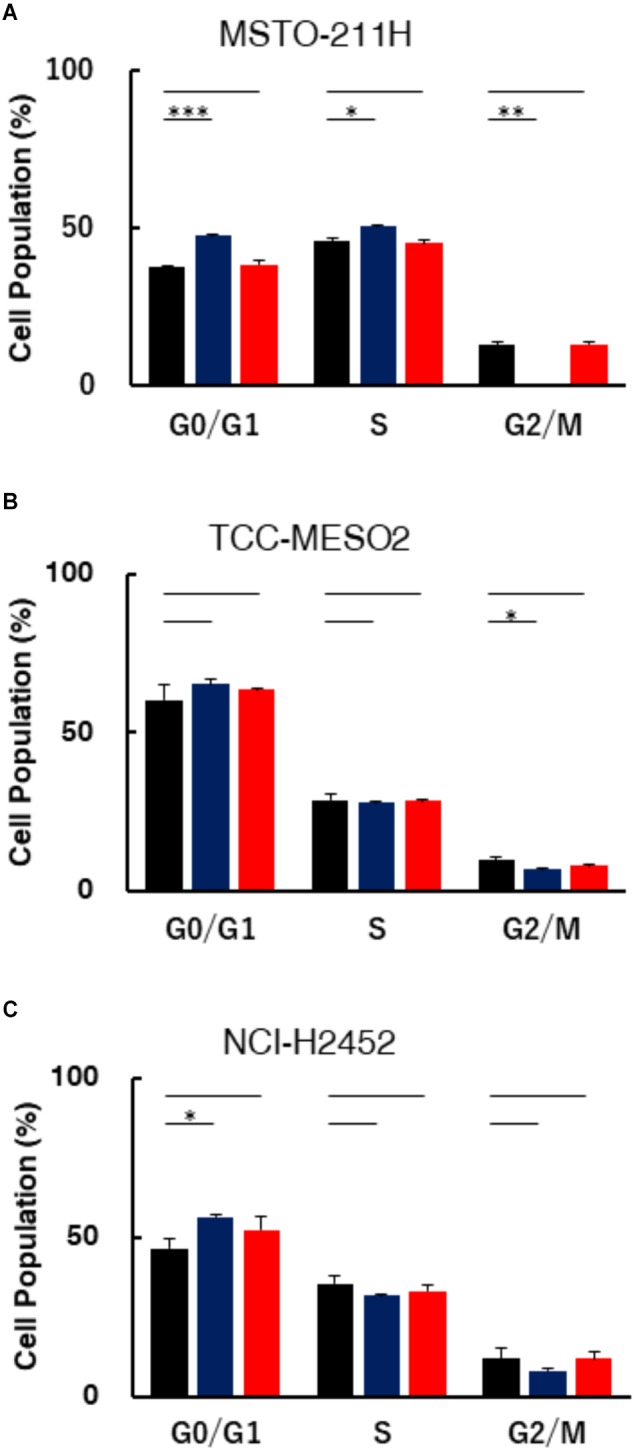
Assessment of DNA content in MPM cells treated with pemetrexed. Treatment with pemetrexed arrested cell cycle in both the pemetrexed-sensitive MSTO-211H **(A)** and TCC-MESO2 **(B)**, and the PMX-resistant NCI-H2452 **(C)** MPM cells. After 6 h of 1 μM pemetrexed treatment, the cells were analyzed for DNA content via PI staining. The percentage of cell populations in the G0/G1, S, and G2/M phases. Black: PBS, Blue: Pemetrexed (PMX) 1 μM, Red: PMX 1 μM + 100 μM hypoxanthine + 16 μM thymidine (HT). The data were shown as means ± *s.d.* From at least three independent experiments (*n* = 3). ^∗^*P* < 0.05, ^∗∗^*P* < 0.01, ^∗∗∗^*P* < 0.001 vs. control by two-tailed Student’s *t*-test.

To characterize the impact of pemetrexed treatment on cell cycle progression, we performed cell cycle analysis in MPM cells fixed and stained with PI (propidium iodide). The DNA content of the cells treated with pemetrexed for 6 h is shown in **Figure 3**, while the data for 24 h is shown in **Supplementary Figure S1**. Cell cycle distributions were determined by flow cytometric analyses of MSTO-211H, TCC-MESO2 and NCI-H2452 cells treated with PBS or 1 μM pemetrexed, which is a relatively high concentration of pemetrexed for a short period of time (6 h). In pemetrexed-treated MSTO-211H cells, we observed a significant accumulation in the G0/G1 phase (47.7 ± 0.4%) of the cell cycle compared with 37.8 ± 0.1% in G1 when treated with PBS control (**Figure 3A**). The treatment of MSTO-211H cells with pemetrexed also induced S phase cell cycle arrest at 50.7 ± 0.1% of the cell population in the presence of pemetrexed as compared to 45.8 ± 1.2% with PBS control. Cell cycle arrest was released when HT solution was added into culture medium (**Figure 3A**). In both pemetrexed-sensitive cell lines, MSTO-211H and TCC-MESO2, the G2/M cell population was decreased after treatment with pemetrexed. MSTO-211H cells in G2/M were changed from 12.8% ± 0.1 to 0.0% and TCC-MESO2 cells in G2/M were altered from 9.5 ± 1.0% to 7.7 ± 0.4% (**Figures 3A,B**). Similarly, we observed statistically significant G1 arrest after treatment with pemetrexed in NCI-H2452 cells (**Figure 3C**). As the treatment with pemetrexed altered cell cycle in MPM cells, we analyzed the levels of proteins involved in cell cycle regulation using Western blot (WB). WB analyses of pRB1, RB1 and p21 did not evidence substantial changes in their levels upon pemetrexed treatment (**Supplementary Figure S2**).

### Expression Level of Pemetrexed Target Molecules

**FIGURE 4:**
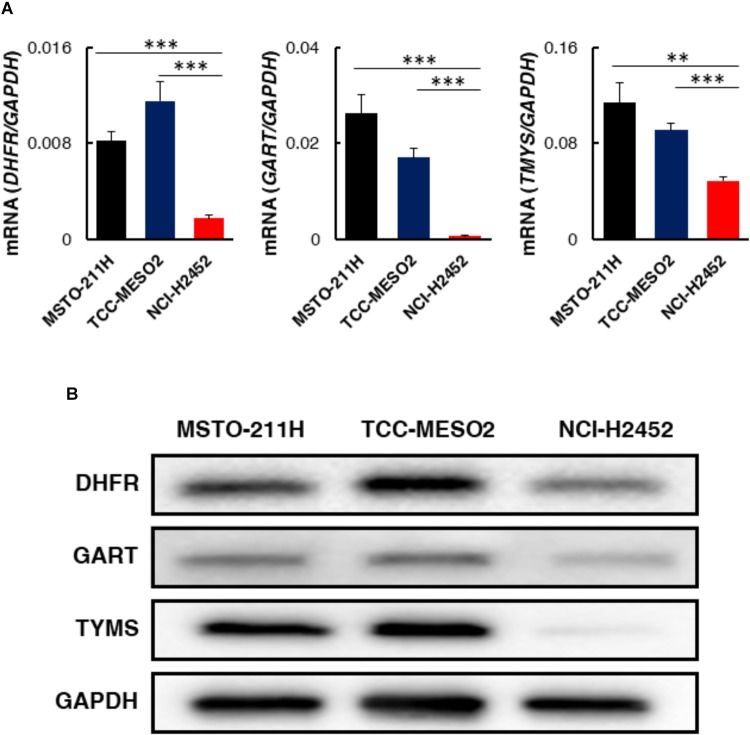
Expression levels of target genes involved in one-carbon metabolism in pemetrexed-sensitive vs. pemetrexed-resistant MPM cells. **(A)** mRNA expression was evaluated by real-time RT-PCR using the ΔΔ*CT* method normalized to the housekeeping gene GAPDH. Asterisks show significant difference from NCI-H2452 cells. Error bars indicate *s.d. (n* = 6). ^∗^*P* < 0.05, ^∗∗^*P* < 0.01, ^∗∗∗^*P* < 0.005 vs. control by two-tailed Student’s *t*-test. **(B)** Representative images of Western blotting (WB) characterizing proteins involved in one-carbon metabolism. Equivalent amounts of proteins from whole-cell lysates were subjected to WB analysis to detect the indicated proteins. GAPDH was used as a loading control.

Pemetrexed and its polyglutamated derivatives mainly inhibit thymidylate synthase (TYMS), dihydrofolate reductase (DHFR), and glycinamide ribonucleotide transformylase (GART), all of which are involved in the *de novo* biosynthesis of thymidine and purine nucleotides (**Supplementary Figure S3**). To investigate the efficacy of one-carbon metabolic pathway inhibition against MPM cells, we investigated whether the expression levels of pemetrexed targets involved in this pathway were different in pemetrexed sensitive vs. resistant MPM cell lines. Several prior studies showed that *TYMS* overexpression is one of the major factors leading to resistance in pemetrexed-resistant cells and that regulation of *DHFR, RFC*, and *FPGS* expression is associated with acquired resistance to antifolate (Zhao and Goldman, 2003). Therefore, we measured the expression level of TYMS, DHFR and GART at the transcriptional and protein levels. Transcriptional expression of genes encoding pemetrexed-target enzymes was measured by RT-PCR (**Supplementary Table S1**). Interestingly, as shown in **Figure 4A**, we observed that DHFR, GART, and TYMS mRNA expression levels were all significantly higher in pemetrexed-sensitive cells as compared to the pemetrexed-resistant NCI-H2452 cells (DHFR: MSTO-211H = 4.7-fold, TCC-MESO2 = 6.5-fold; GART: MSTO-211H = 35.5-fold, TCC-MESO2 = 23.0-fold; TYMS: MSTO-211H = 2.3-fold, TCC-MESO2 = 1.9-fold). To confirm the mRNA results, we analyzed protein levels by WB. Representative images of WB results are shown in **Figure 4B** and quantitative analysis is shown in **Supplementary Figure S4**. Differences in DHFR protein levels in PMX-sensitive MSTO-211H and TCC-MESO2 cells as compared to PMX-resistant NCI-H2452 cells were not statistically significant (**Supplementary Figure S4**, *P* = 0.055). Consistent with the mRNA expression data, the expression of GART (MSTO-211H: 3.4-fold, TCC-MESO2: 3.3-fold), and TYMS proteins (MSTO-211H: 71.9-fold, TCC-MESO2: 81.4-fold) was found to be higher in pemetrexed-sensitive cells as compared to the pemetrexed-resistant NCI-H2452 cells (**Supplementary Figure S4**). Relative levels of GAPDH protein were not changed in MSTO-211H and TCC-MESO2 as compared to NCI-H2452 cells (**Supplementary Figure S4**). The lower level of pemetrexed target molecules in the pemetrexed-resistant NCI-H2452 cells as compared to the sensitive cells suggests that the one-carbon metabolic pathway is altered and that the resistant cells may survive by bypassing folate related pathways.

### Glycolytic Activities Are Not Altered in MPM Cells After Treatment With Pemetrexed

The expression level of enzymes in pemetrexed-sensitive cells was different from resistant cells, suggesting that metabolic profiles could be altered in cells sensitive or resistant to pemetrexed treatment. In cancer cells, the Warburg effect, whereby glucose is consumed with secretion of lactate despite abundant oxygen availability, has been recognized since the 1930s (Vander Heiden et al., 2009; Lunt and Vander Heiden, 2011; Soga, 2013). We hypothesized that glycolytic activity would be correlated with pemetrexed sensitivity. To test this idea, we measured the glucose-induced ECAR, an indicator of lactate production, and the oxygen consumption rate (OCR), an indicator of oxidative phosphorylation (OXPHOS), using a flux analyzer. Basal levels of ECAR at the beginning of measurements, which indicated non-glycolytic acidification, were low in all MPM cells (**Supplementary Figure S5**). Equivalent ECAR was observed in MPM cells both pre- and post-treatment with an ATPase inhibitor oligomycin to induce maximum cellular glycolytic capacity (**Supplementary Figure S5**). At the final step, the addition of 2-deoxy-D-glucose (2DG), an inhibitor of glycolysis, completely shut down extracellular acidification (**Supplementary Figure S5**). ECAR was not statistically different in sensitive or resistant MPM cells either with or without pemetrexed (**Supplementary Figure S5**).

### Alterations in Metabolic Pathways in the Response to Pemetrexed Treatment

**FIGURE 5:**
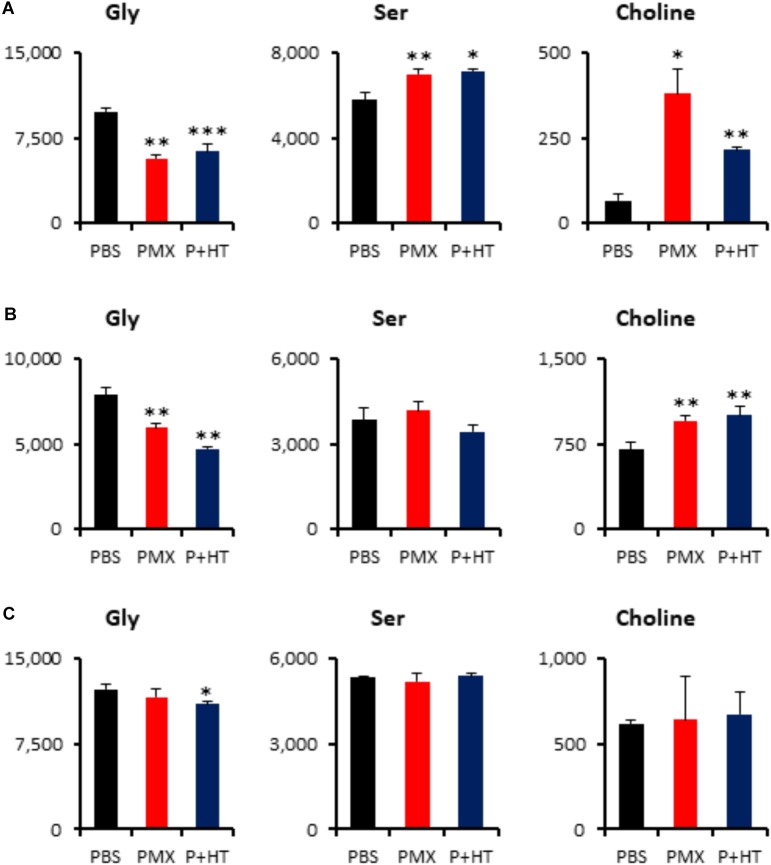
Quantification of metabolites involved in the one-carbon pathway. Intracellular concentration (pmol/million cells) of key metabolites involved in the one-carbon metabolic pathway after treatment with pemetrexed is shown. Error bars indicate *s.d. (n* = 3). ^∗^*P* < 0.05, ^∗∗^*P* < 0.01, ^∗∗∗^*P* < 0.001 vs. control by Welch *t*-test. Total metabolites were extracted with methanol from MSTO-211H **(A)**, TCC-MESO2 **(B),** or NCI-H2452 **(C)** cells treated with PBS (black), 1 μM PMX (red) or 1 μM PMX + HT (blue) for 6 h. Representative metabolites such as glycine (Gly), serine (Ser), and choline are shown.

To investigate whether treatment of MPM cells with pemetrexed affects global cancer metabolism, we employed metabolomics analysis. Intracellular metabolites were extracted with methanol and analyzed using capillary electrophoresis time-of-flight mass spectrometry (CE-TOFMS) (Soga et al., 2003) and the profile of 117 quantified metabolites is shown in **Supplementary Table S2**. Principal component analysis (PCA) and heatmap analysis of metabolomics datasets within the indicated cell lines and culture conditions are shown in **Supplementary Figure S6**. The amount of adenosine triphosphate (ATP), which is the molecular unit of currency of intracellular energy transfer, was not changed in any of the three tested cell lines after pemetrexed treatment for 6 h (**Supplementary Table S2**). Thymidine was not detected in MPM cells under any experimental conditions (**Supplementary Table S2**). With respect to one-carbon metabolism, glycine was significantly decreased in pemetrexed-sensitive cells after treatment with pemetrexed, but not in the resistant NCI-H2452 cells (**Figure 5**). On the other hand, choline accumulated in pemetrexed-sensitive cells after pemetrexed treatment, but not in resistant NCI-H2452 cells (**Figure 5**). Although the folate metabolic pathway is linked with the methionine cycle and polyamine biosynthesis pathways (**Supplementary Figure S3**), intermediate metabolites were not changed by pemetrexed treatment in both pemetrexed-sensitive and resistant cells (**Supplementary Table S2**).

**FIGURE 6:**
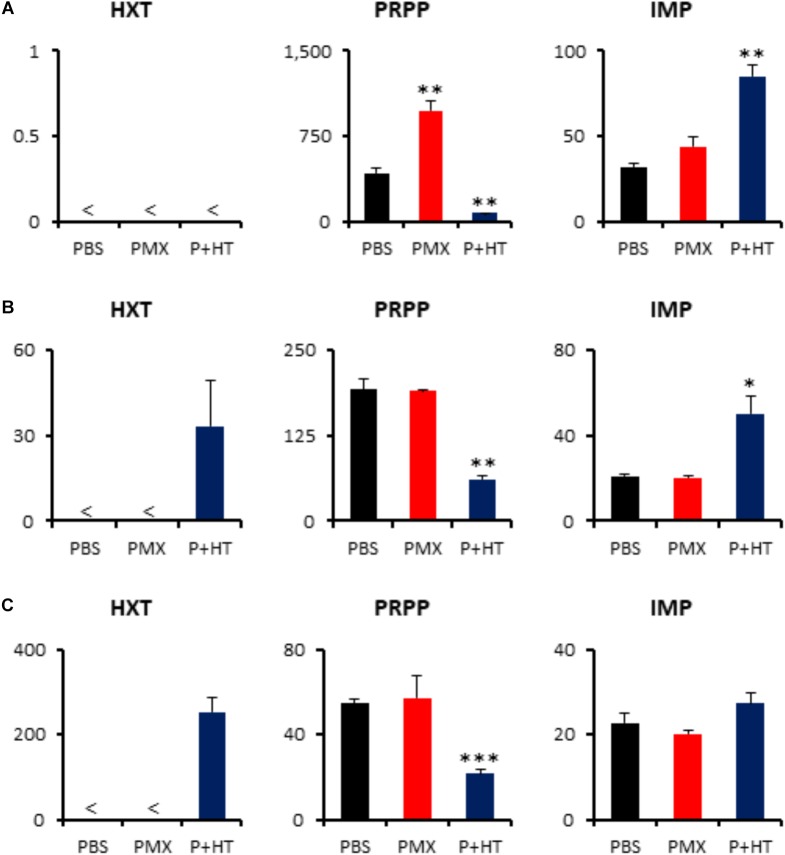
Quantification of metabolites involved in purine salvage pathway. Intracellular concentration (pmol/million cells) of key metabolites involved in the purine salvage pathway after treatment of pemetrexed is shown. Error bars indicate *s.d. (n* = 3) and < indicates the metabolite was not detected (N.D.). ^∗^*P* < 0.05, ^∗∗^*P* < 0.01, ^∗∗∗^*P* < 0.001 vs. control by Welch *t*-test. Total metabolites were extracted with methanol from MSTO-211H **(A)**, TCC-MESO2 **(B)**, or NCI-H2452 **(C)** cells treated with PBS (black), 1 μM PMX (red) or 1 μM PMX + HT (blue) for 6 h. Representative metabolites including hypoxanthine (HXT), PRPP, and IMP are shown.

The addition of exogenous hypoxanthine and thymidine is sufficient to provide the end product of the pemetrexed block and to permit DNA synthesis for cell division (**Figure 2**). Although intracellular thymidine was not detected in MPM cells under any experimental condition (**Supplementary Table S2**), intracellular hypoxanthine was detected in TCC-MESO2 and NCI-H2452 cells (**Figure 6**). Metabolome analysis revealed that HT treatment with pemetrexed significantly decreased the levels of PRPP in both pemetrexed-sensitive and pemetrexed-resistant cell lines (**Figure 6**). On the other hand, IMP, which is a product of the purine salvage pathway, was increased in the pemetrexed-sensitive MPM cells treated with pemetrexed + HT solution (**Figures 6A,B**), but not in resistant NCI-H2452 cells (**Figure 6C**).

### MTAP Expression and MTA Effect for MPM Cell Survival

**FIGURE 7:**
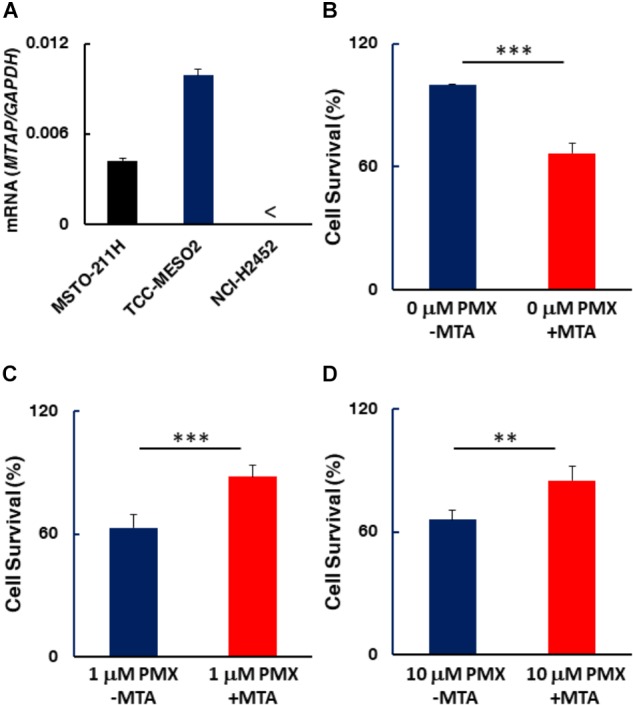
MTAP expression in MPM cells and effect of MTA on MPM cell survival. **(A)** *MTAP* mRNA expression was evaluated by real-time RT-PCR using the ΔΔCT method normalized to the housekeeping gene GAPDH. < indicates not detected (N.D.). Error bars indicate *s.d. (n* = 6). **(B–D)** Addition of 5′-deoxy-5′-(methylthio)-adenosine (MTA) represses drug-resistant cells. NCI-H2452 cell survival (%) was measured in the presence of 0.1 mM MTA co-treated without **(B)** or with either 1 μM **(C)** or 10 μM pemetrexed **(D)**. The data are shown as the mean ± *s.d.* (*n* = 6). Error bars represent one *s.d.*^∗^*P* < 0.05, ^∗∗^*P* < 0.01, ^∗∗∗^*P* < 0.005 vs. control by two-tailed Student’s *t*-test.

A previous study has shown homozygous deletion of *CDKN2A* and co-deletion of the *MTAP* gene in the majority of clinical cases of MPM (Illei et al., 2003). To determine the status of MTAP expression in MPM cell lines used here, we quantified *MTAP* mRNA expression levels by RT-PCR. MTAP gene expression was detected in the pemetrexed-sensitive MSTO-211H and TCC-MESO2 cells, but not in pemetrexed-resistant NCI-H2452 cells (**Figure 7**). Given our observation that pemetrexed treatment with HT solution significantly decreased the levels of PRPP in all MPM cells whereas IMP was upregulated in pemetrexed-sensitive but not pemetrexed-resistant cells (**Figure 6**), we hypothesized that MTA changed the pemetrexed sensitivity of MPM cells. To test this, we added MTA to the culture medium and measured survival of the NCI-H2452 pemetrexed-resistant cell line treated with 0, 1, or 10 μM pemetrexed (**Figure 7**). As predicted, the addition of MTA to the culture media of the drug-resistant cell line, NCI-H2452, resulted in a growth-repressive phenotype, as reflected by decreased cell survival (**Figure 7B**). In contrast, the addition of MTA to the culture media of NCI-H2452 cells treated with pemetrexed resulted in a more pemetrexed-resistant phenotype (**Figures 7C,D**). These results indicate that the vulnerability to targeting of the one-carbon metabolic pathway in MPM cells may be dependent on the purine biosynthesis pathway.

## Discussion

Antifolate is feasible chemotherapeutic option to treat MPM in the clinic. Most antifolate drugs including pemetrexed inhibit DHFR, TYMS and GART molecules involved in purine and pyrimidine biosynthesis. There still exists a need to develop predictive metabolic biomarkers that would allow stratification of patients for effective therapy with pemetrexed targeting the one-carbon metabolism pathway. In this paper, we found that MPM cell lines could be categorized into two groups based on their sensitivity to pemetrexed treatment. Hypoxanthine and thymidine, which are end products of the pemetrexed-targeted metabolic pathway, are sufficient to cancel the cytotoxic effects of pemetrexed. Moreover, we found that the expression of pemetrexed-targeted enzymes was lower in pemetrexed-resistant MPM cells than in pemetrexed-sensitive cells on a molecular basis. Metabolomic analysis revealed that glycine and choline, which are involved in one carbon metabolism, were altered after treatment in pemetrexed-sensitive MPM cells, but not in resistant cells. In addition, the supplementation of HT induced higher concentrations of IMP in pemetrexed-sensitive MPM cells. Finally, we showed that the addition of MTA altered pemetrexed-sensitivity in MTAP-negative NCI-H2452 cells, indicating that the nucleic acid biosynthesis pathway is important for predicting the efficacy of pemetrexed in MPM cells.

The lower level of pemetrexed target molecules in the pemetrexed-resistant NCI-H2452 cells suggests that they may survive by bypassing folate related pathways (**Figure 4**). In addition, the level of intracellular glycine was decreased after treatment with pemetrexed in the sensitive MSTO-211H and TCC-MESO2 cells (**Figure 5**). In contrast to glycine, serine accumulated only in the MSTO-211H cells (**Figure 5**). Accumulating evidence indicates that serine and glycine metabolism is important for cancer growth and proliferation (Jain et al., 2012; Zhang et al., 2012; Kim et al., 2015; Ducker et al., 2017). The enzyme serine hydroxymethyltransferase (SHMT) catalyzes the conversion of serine and tetrahydrofolate (THF) into glycine and 5,10-methylene-THF (**Supplementary Figure S3**). Similarly, the enzyme glycine decarboxylase (GLDC) catalyzes the conversion of glycine and tetrahydrofolate (THF) into ammonia, carbon dioxide and 5,10-methylene-THF (**Supplementary Figure S3**). Although serine is the predominant source of one-carbon units in cancer cells (Labuschagne et al., 2014), the level of glycine was more dramatically down-regulated than serine (**Figure 5**). Glycine consumption and synthesis are correlated with rapid cancer cell proliferation and glycine metabolism supports *de novo* purine nucleotide biosynthesis. Moreover, the metabolic enzyme GLDC is critical for tumor-initiating cells in non-small cell lung cancer (NSCLC) (Zhang et al., 2012). Further investigation would be needed to characterize in greater detail the molecular mechanisms that control glycine metabolism in MPM cells.

Several studies have suggested that TYMS is a predictive biomarker for the use of pemetrexed in NSCLC (Bukhari and Goudar, 2013). However, these associations are controversial. Prior studies showed that TYMS overexpression was one of the major factors leading to resistance in pemetrexed-resistant cells (Zhao and Goldman, 2003). However, our study demonstrated the opposite results (**Figure 4**). This suggests that differences in degree of pemetrexed-resistance and patterns of resistance may be attributed to multiple factors that include drug transport and polyglutamination as well as changes in cellular folate pools that must be assessed and considered in pemetrexed-resistant MPM cells. A recent report showed that the balance between folic acid uptake, activation and utilization plays a crucial role in the response to pemetrexed-based chemotherapy and prognosis in MPM (Mairinger et al., 2017). We observed that the addition of HXN partially rescued PMX cytotoxicity in TCC-MESO2 cells but had no effect in MSTO-211H and NCI-H2452 cell lines (**Figure 2**). With regards to the difference between MSTO-211H and TCC-MESO2 cells in the treatment of PMX + HXN, we speculate that pemetrexed mainly inhibits the pyrimidine biosynthesis pathway in MSTO-211H cells and inhibits both pyrimidine and purine biosynthesis pathways in TCC-MESO2 cells. Our results showed that the combination of hypoxanthine plus thymidine completely rescued the growth repression induced by pemetrexed (**Figure 2**). We reasoned that the treatment with HT solution might induce the mRNA expression level of PMX-target genes involved in one-carbon metabolism. However, the mRNA levels in both PMX-sensitive vs. PMX-resistant MPM cells was almost equivalent even after treatment with HT solution (**Supplementary Figure S7**). Therefore, the measured concentrations of hypoxanthine and thymidine in the serum or within the tumor tissue of MPM patients may directly correlate with non-responsiveness for pemetrexed treatment. To test this idea, we will need to analyze nucleic acid metabolites in clinical samples for these and other potential biomarkers to predict therapeutic response in MPM.

## Author Contributions

HM, JN, SU, AO, MT, TS, and KT designed the study and contributed to analysis and interpretation of data. YS and HM wrote the initial draft of the manuscript. All other authors have contributed to data collection and interpretation, and critically reviewed the manuscript. All authors approved the final version of the manuscript and agree to be accountable for all aspects of the work.

## Conflict of Interest Statement

The authors declare that the research was conducted in the absence of any commercial or financial relationships that could be construed as a potential conflict of interest.

## Abbreviations

ECAR: extracellular acidification rate
IMP: inosine monophosphate
MPM: malignant pleural mesothelioma
MTA: 5-methylthioadenosine
MTAP: methylthioadenosine phosphorylase
PRPP: phosphoribosyl diphosphate
