# Supplementary figures and images for: Metabolic Characterization of Antifolate Responsiveness and Non-responsiveness in Malignant Pleural Mesothelioma Cells
Source: Front Pharmacol. 2018 Oct 12;9:1129. doi: 10.3389/fphar.2018.01129 (PMC6194193; doi:10.3389/fphar.2018.01129)

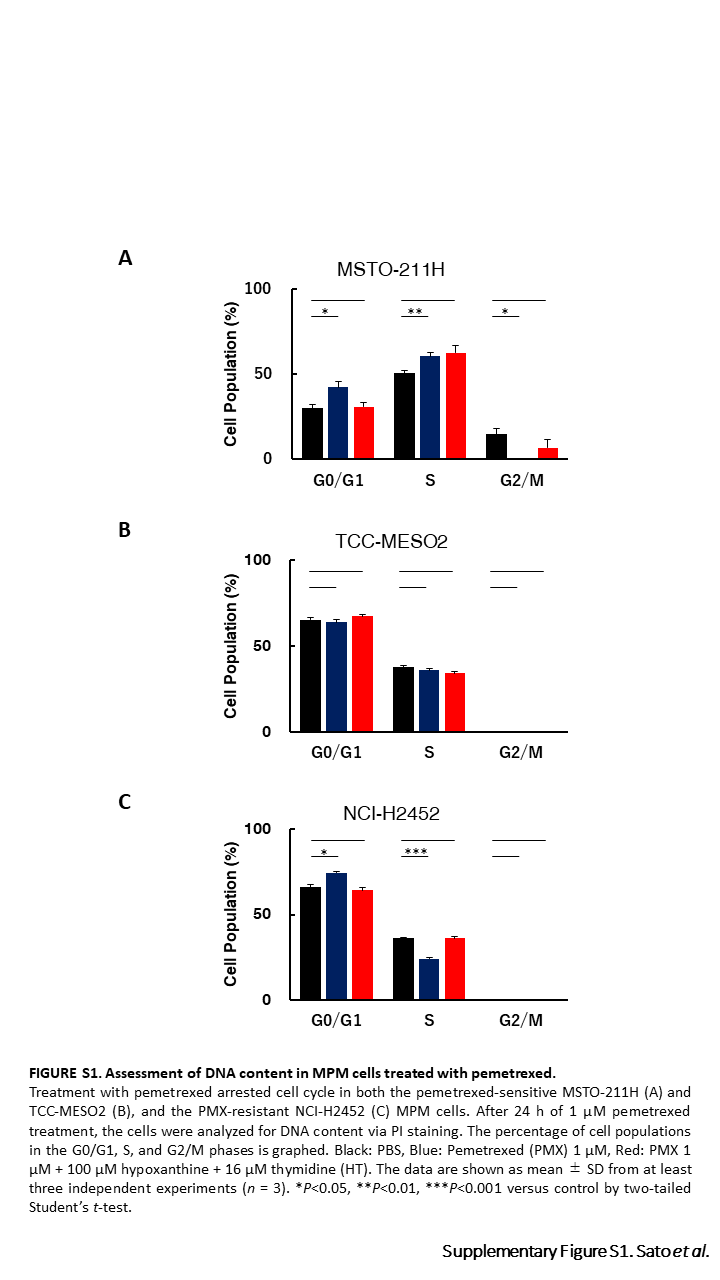

Supplement: Supplementary file 1 [file Image_1.TIF]

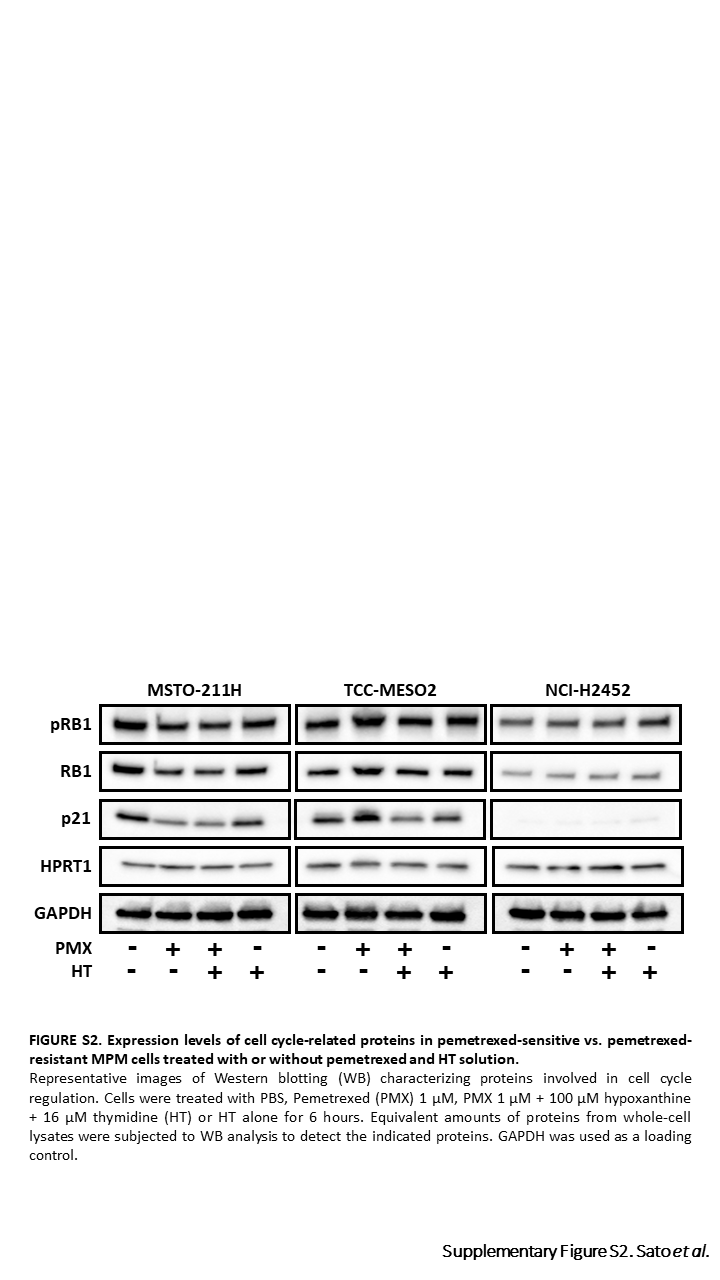

Supplement: Supplementary file 2 [file Image_2.TIF]

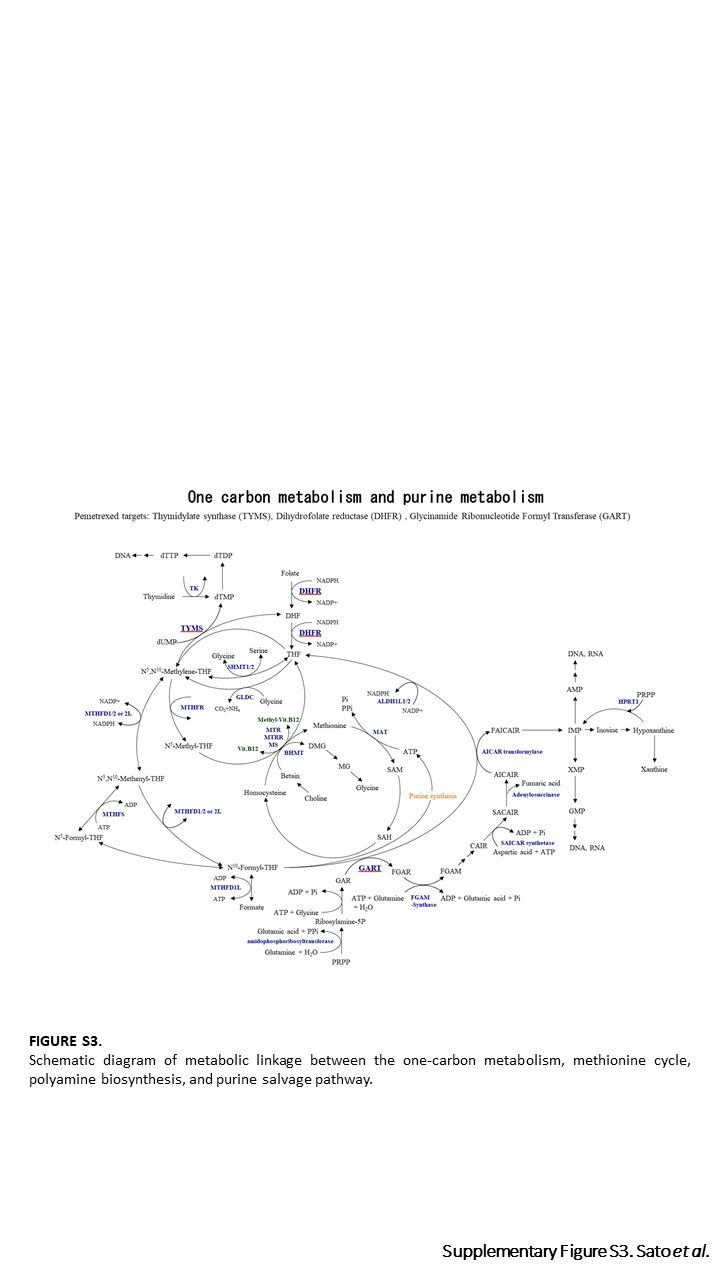

Supplement: Supplementary file 3 [file Image_3.TIF]

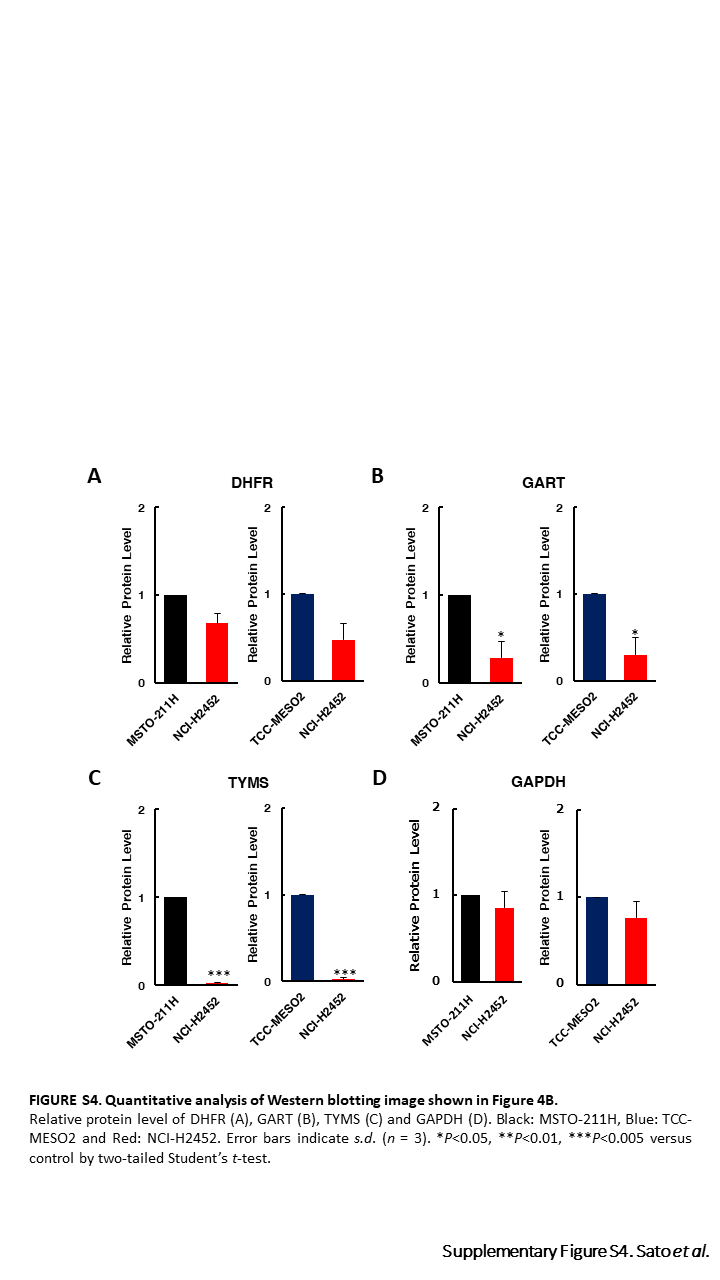

Supplement: Supplementary file 4 [file Image_4.TIF]

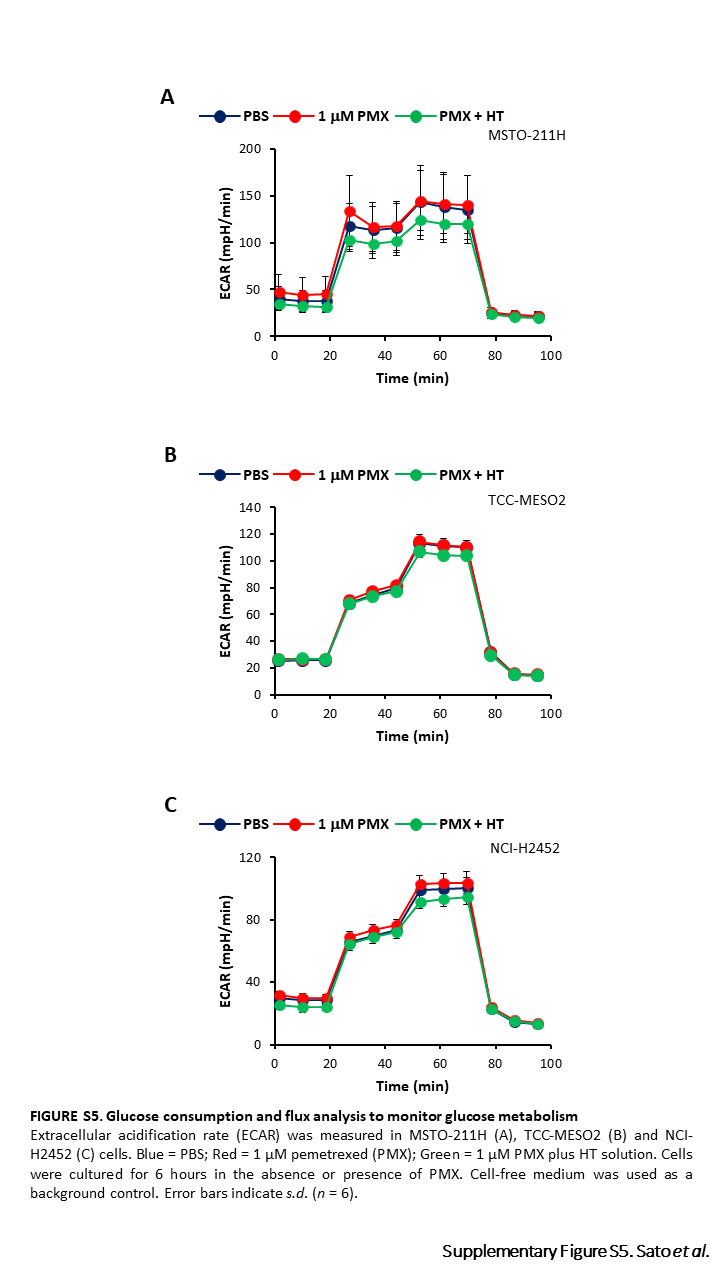

Supplement: Supplementary file 5 [file Image_5.TIF]

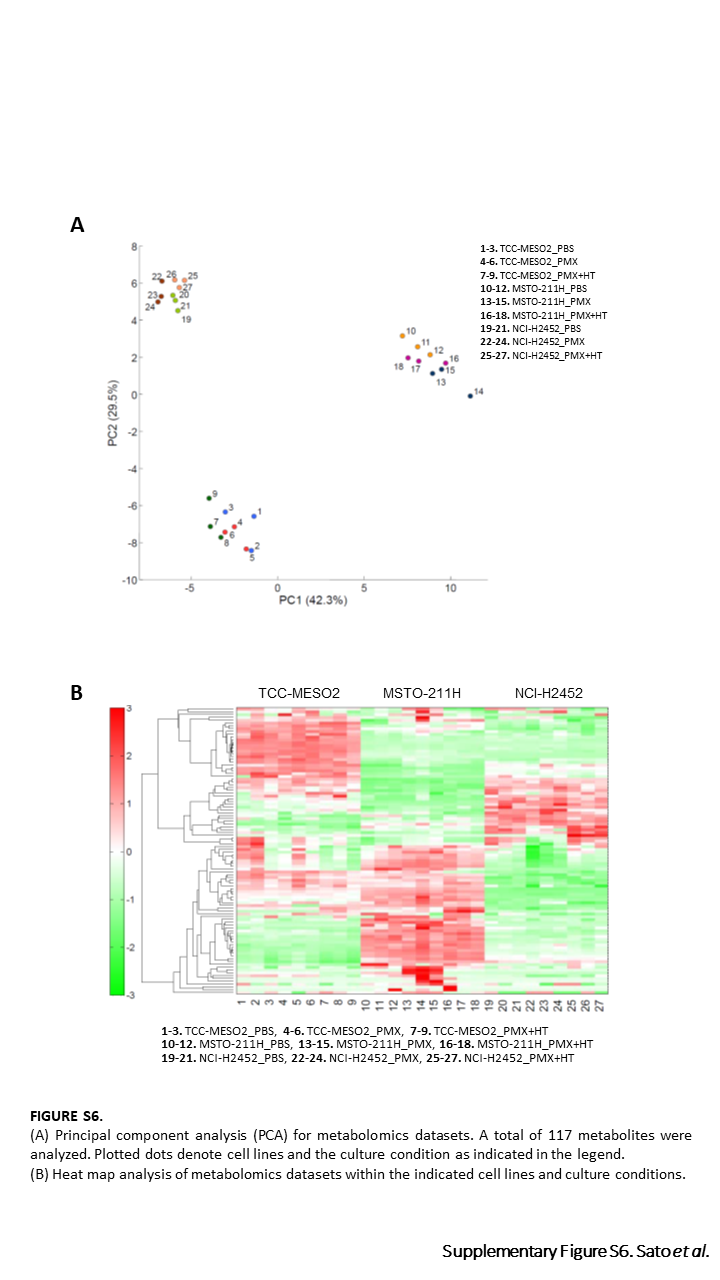

Supplement: Supplementary file 6 [file Image_6.TIF]

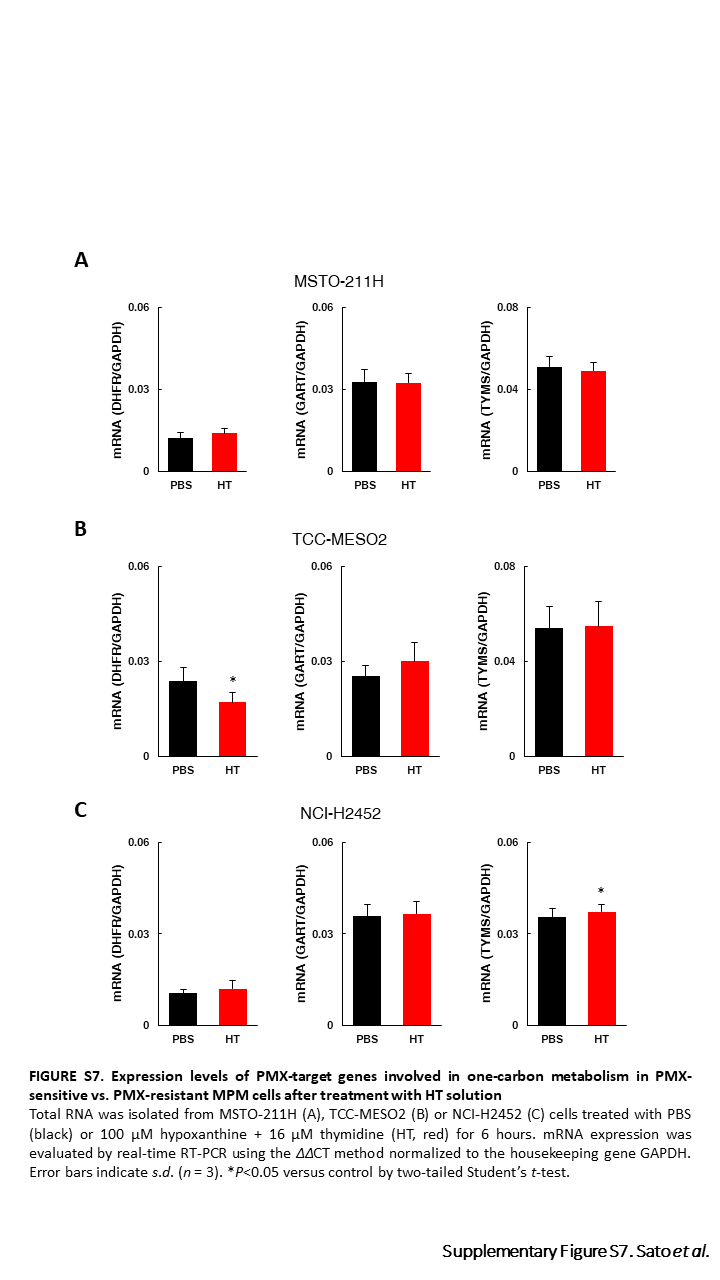

Supplement: Supplementary file 7 [file Image_7.TIF]
